# *TRPV1* variants impair intracellular Ca^2+^ signaling and may confer susceptibility to malignant hyperthermia

**DOI:** 10.1038/s41436-018-0066-9

**Published:** 2018-06-21

**Authors:** Fabien Vanden Abeele, Sabine Lotteau, Sylvie Ducreux, Charlotte Dubois, Nicole Monnier, Amy Hanna, Dimitra Gkika, Caroline Romestaing, Lucile Noyer, Matthieu Flourakis, Nolwenn Tessier, Ribal Al-Mawla, Christophe Chouabe, Etienne Lefai, Joël Lunardi, Susan Hamilton, Julien Fauré, Fabien Van Coppenolle, Natalia Prevarskaya

**Affiliations:** 1 Inserm U1003, Laboratory of Excellence, Ion Channels Science and Therapeutics, Equipe Labélisée par la Ligue Nationale Contre le Cancer, SIRIC ONCOLille, Université des Sciences et Technologies de Lille, Villeneuve d’Ascq, 59656 France; 2 Université de Lyon, Lyon, Cedex 07 France; 3 Univ-Lyon, CarMeN laboratory, Inserm U1060, INRA U1397, Université Claude Bernard Lyon1, INSA Lyon, Hospital Cardiology, IHU OPERA Cardioprotection, B13 Building, Lyon East, 59 Boulevard Pinel, Fr-69500 Bron, France; 4 School of Biomedical Sciences, University of Leeds, Leeds, LS2 9JT UK; 5 Laboratoire de Biochimie Génétique et Moléculaire, IBP, CHU Grenoble Alpes, F-38000 Grenoble, France; 6 Department of Molecular Physiology and Biophysics, Baylor College of Medicine, Houston, Texas 77030 USA; 7 Université de Lyon, UMR 5023 Ecologie des Hydrosystèmes Naturels et Anthropisés, Université Lyon 1, ENTPE, CNRS, 6 rue Raphaël Dubois, 69622 Villeurbanne, France; 8 INSERM U1216, F-38000 Grenoble, France; 9 Univ. Grenoble Alpes, Grenoble Institut des Neurosciences, GIN, F-38000 Grenoble, France

**Keywords:** TRP channel, TRPV1, Calcium, Hereditary disease, Malignant hyperthermia

## Abstract

**Purpose:**

Malignant hyperthermia (MH) is a pharmacogenetic disorder arising from uncontrolled muscle calcium release due to an abnormality in the sarcoplasmic reticulum (SR) calcium-release mechanism triggered by halogenated inhalational anesthetics. However, the molecular mechanisms involved are still incomplete.

**Methods:**

We aimed to identify transient receptor potential vanilloid 1 (*TRPV1*) variants within the entire coding sequence in patients who developed sensitivity to MH of unknown etiology. In vitro and in vivo functional studies were performed in heterologous expression system, *trpv1*^−/−^ mice, and a murine model of human MH.

**Results:**

We identified *TRPV1* variants in two patients and their heterologous expression in muscles of *trpv1*^−/−^ mice strongly enhanced calcium release from SR upon halogenated anesthetic stimulation, suggesting they could be responsible for the MH phenotype. We confirmed the in vivo significance by using mice with a knock-in mutation (Y524S) in the type I ryanodine receptor (Ryr1), a mutation analogous to the Y522S mutation associated with MH in humans. We showed that the TRPV1 antagonist capsazepine slows the heat-induced hypermetabolic response in this model.

**Conclusion:**

We propose that TRPV1 contributes to MH and could represent an actionable therapeutic target for prevention of the pathology and also be responsible for MH sensitivity when mutated.

## Introduction

The role of Ca^2+^ as the main regulatory and signaling molecule in skeletal muscle contraction is well described, and  pathogenic variants in several genes encoding Ca^2+^ signaling and handling molecules are responsible for various myopathies, yet understanding of the molecular mechanisms involved is still incomplete.^1–3^

Altered regulation of Ca^2+^ release is a key contributor to the pathophysiology of core myopathies such as central core disease (CCD, OMIM 117000), as well as in the hypermetabolic response associated with anesthesia-induced malignant hyperthermia (MH, OMIM 145600), a triggered muscle disease.^4^ These pathologies have so far been mainly linked to mutations in the *RYR1* gene, which encodes the main intracellular Ca^2+^-release channel of skeletal muscle.^5,6^ Generally, these mutations lead to a hypersensitivity of the RyR1 channel to activation by a wide range of triggers, including caffeine, halothane, and Ca^2+^, or to a decrease in voltage-induced activation.^6–8^ The enhanced intracellular Ca^2+^ results in abnormal skeletal muscle metabolism manifesting as activation of muscle contraction mediated by the binding of Ca^2+^ on troponin, thereby allowing the movement of tropomyosin on actin filaments. This abnormal skeletal muscle metabolism is also characterized by increased oxygen consumption, adenosine triphosphate (ATP) hydrolysis, and heat production.^9,10^ Although linkage to the *RYR1* gene is shown for more than 50% of all MH cases, mutations in two other genes were associated to MH: *CACNA1S* encoding the L-type plasma membrane channel regulating RyR1, and *STAC3*, a small adaptor protein interacting with both channels.

The search for a new candidate potentially involved in MH led us to the TRPV1 channel. To date, although playing a widely accepted role in nociception, this channel is not clearly linked to any hereditary disease.^11^ Efforts have been mainly focused on its implication in chronic pain syndromes,^11^ however, the latest available data suggest that TRPV1, as a highly Ca^2+^-permeable channel, also plays an important role in the physiology of skeletal muscle.^12^ Indeed, after initial discovery as a neuronal sensory channel, TRPV1 was subsequently found in a few non-neuronal tissues, including skeletal muscle, and recent studies have clearly highlighted its functional role as a sarcoplasmic reticulum (SR) Ca^2+^ channel.^13,14^ Interestingly, TRPV1 has recently been shown to be sensitive to volatile anesthetics in neurons.^15,16^ However, the potential implication of TRPV1 channel in the mechanism leading to altered regulation of Ca^2+^ signaling in the pathophysiology of MH has not yet been investigated.

Here, using complementary approaches and models, we identify and functionally characterize human variants of the TRPV1 channel that confer muscle sensitivity to anesthetics exposure. Our results suggest that TRPV1 plays a critical role in the aberrant Ca^2+^ homeostasis in MH.

## Material and methods

### Patients

The 28 patients tested were referred to the laboratory for genetic screening of the *RYR1* (OMIM 180901) gene in the context of malignant hyperthermia (MH, OMIM 145600). In all cases a familial history of MH during anesthesia was reported. They were all tested for MH susceptibility with an in vitro contracture test (IVCT) according to recommendation of the European Malignant Hyperthermia Group (emhg.org). All were sensitive for halothane but not for caffeine, hence diagnosed as MHS(h).

### Genetic analysis

Blood samples used for genetic screening were obtained after a written informed consent was signed by patients according to the French regulation for genetic studies. For each patient, the 15 coding exons of the*TRPV1* gene were amplified from genomic DNA (OMIM 602076, transcript: NM_018727.5, protein: 839 AA, Q8NER1, primer sequences available upon request). The analysis of the entire coding sequence of*TRPV1* was performed by direct sequencing on an ABI 3130 DNA sequencer (PE Applied Biosystems®, Foster City, CA, USA). Variations in the sequence were compared with databases (National Center for Biotechnology Information's dbSNP, 1000 Genomes, and the Exome Variant Server), and selected for the study when allele frequency was less than 1%.

### Cells

HEK-293 cells (American Type Culture Collection) were cultured in Dulbecco’s minimal essential medium (DMEM) and GlutaMAX (Invitrogen, Life Technologies) supplemented with 10% fetal bovine serum (Sigma-Aldrich). Cells were maintained in 5% CO_2_, 95% air at 37 °C in a humidified incubator. Cells (50–60% confluency) were transiently transfected by either 1 μg (in 35-mm dishes) of plasmid (transfecting either TRPV1 wild-type or mutants plus pEGFP-N1 at a 5:1 ratio (pEGFP-N1 was used as a positive control of transfection)) using X-tremeGENE 9 DNA Transfection Reagent (Roche Diagnostics, France) as described by the manufacturer. Cells transfected with 1 µg of pEGFP-N1 alone were used as control (CTL) condition. Cells were mycoplasma-free.

### Cloning

The coding sequence of human *TRPV1* was amplified from the pCAGGSM2-IRES-GFP-TrpV1 vector (a gift from B. Nilius, Leuven, Belgium) and cloned into the pcDNA5/FRT vector (Invitrogen, LifeTechnologies). TRPV1 mutants were obtained using in vitro mutagenesis (QuikChange Site-directed Mutagenesis kit, Agilent Technologies-Stratagene products) and the following primer pairs: T612M ccgtctgagtccatgtcgcacaggtgg / ccacctgtgcgacatggactcagacgg, R722C gtgagggcgtcaagtgcaccctgagcttc/gaagctcagggtgcacttgacgccctcac, and V1394del cacctgcgagaagtcggtgctggagg/cctccagcaccgacttctcgcaggtg. TRPV1 wild-type and mutants were subcloned into the pmCherry-C1 vector as an EcoRI-KpnI fragment. All long polymerase chain reactions (PCRs) mentioned above were carried out with the High Fidelity Phusion DNA Polymerase (Finnzymes) and all constructs were verified by sequence analysis.

### Ethics statement

All experiments were performed in accordance with the guidelines of the French Ministry of Agriculture (87/848) and of the European Community (86/609/EEC). They were approved by the local animal ethics committee of Rhône-Alpes, approval number 692660602.

### Indirect calorimetry

Wild-type (WT) and YS (heterozygous RyR1^Y524S/WT^ knock-in) male mice (on C57/B6 background, 6–10 weeks old) were weighed and injected with 5 mg/kg (IP) capsazepine dissolved in 2% DMSO and 10% Tween 20 in sterile saline (vehicle) or with vehicle alone. Ten minutes after injection, mice were placed individually into indirect calorimetry chambers (Oxymax System, Columbus Instruments) contained with a temperature controlled environment chamber set at 37 °C. Maximum oxygen consumption (VO_2_, mL/kg/min) was monitored for 15 min before mice were removed from the chambers. All procedures were approved by the Animal Care Committee at Baylor College of Medicine.

### Isolation of muscle fibers

Single skeletal muscle fibers were isolated from the flexor digitorum brevis muscles of 4 to 8-week-old wild-type (C57BL6J from Charles Rivers Laboratories) or TRPV1^−/−^ (from Jackson Laboratories) male mice. Mice were killed by cervical dislocation. Muscles were removed and treated with type 1 collagenase (45–60 min at 37 °C) in the presence of Tyrode as external solution. Single fibers were then obtained by triturating muscles within the experimental chamber. Cells were mounted into a glass bottom dish. Fibers were bathed in the presence of Fluo-4 AM (5 µM) during 30 min. Cells were then washed with Tyrode solution.

### In vivo transfection

Expression of *TRPV1* variants by electroporation was performed in the flexor digitorum brevis of 4 to 8-week-old*trpv1*^−/−^ male mice using a previously described procedure.^17–19^ Mice were anesthetized by isoflurane inhalation (5 min, 3% in air, air flow at 300 ml.min^−1^) using a commercial delivery system (Univentor 400 Anaesthesia Unit, Uninventor, Zejtun, Malta). During anesthesia, 25 µl of a solution containing 2 mg/ml hyaluronidase dissolved in sterile saline was injected into the footpads of each hind paw. Mice recovered from anesthesia. Forty minutes later, mice were reanesthetized by isoflurane inhalation. First, 20 µl of plasmid DNAs (mcherry-hTRPV1, mcherry-T612M, or mcherry-N394del) were injected into the footpads of the animal (1.5 mg/ml in standard Tyrode solution). Then, 10 min after plasmid injection, two gold-plated stainless steel acupuncture needles connected to the electroporation apparatus were inserted under the skin, near the proximal and distal portion of the foot, respectively. Twenty pulses of 110 V/cm amplitude and 20 ms duration were delivered at a 2-Hz frequency by a BTX ECM 830 square wave pulse generator (Harvard Apparatus, Holliston, MA, USA). Mice recovered from anesthesia and experimental observations and measurements were carried out 8 days later.

### Immunostaining

Fluorescence of immunostaining was measured on a Zeiss LSM 5 Exciter laser scanning confocal microscope.

### Confocal Ca^2+^ imaging and image analysis

Unless otherwise specified, imaging was achieved on a Zeiss LSM 5 Exciter laser scanning confocal microscope. The microscope was equipped with a 63× oil immersion objective (numerical aperture (NA) = 1.4). Fluo-4 was excited with 488-nm argon laser. The emitted fluorescent light was measured at wavelengths >505 nm. Because Ca^2+^ responses to TRPV1 agonists revealed slow kinetics, images (512/512 pixels) were taken with a 5- or 15-second interval. Fluorescence of regions of interest was normalized to baseline fluorescence (F0). Experiments realized on HEK cells were performed using the membrane-permeable Ca^2+^-sensitive dye fura-2AM, as detailed previously.^20^ Each experiment was repeated three times (field of 35 to 45 cells) and representative experiments are presented (mean ± SE).

### Reagents and preparation of anaesthetics

Capsaicin, capsazepine, and halothane were purchased from Sigma-Aldrich (Saint-Quentin Fallavier, France); isoflurane from Laboratoires Belamont (Neuilly Sur Seine, France). Other reagents were purchased from Sigma-Aldrich.

### Biotinylation

Cells were transfected with 2 µg of each construct. Control experiments were performed by transfecting the empty vectors. Forty-eight hours after transfection, cells were subjected to cell-surface biotinylation and precipitated after lysis with neutravidin–agarose beads (Pierce Rockford, IL, USA) as described in.^21^ Anti-TRPV1 antibody (1/500, Santa Cruz) and anti-calnexin (1/2000, Millipore) were used.

### Data analysis

Results were expressed as the means ± S.E.M. Normality was check using the D’Agostino–Pearson test. Normally distributed data are expressed as mean ± S.E.M and statistical comparison were made using Student’s *t* test. Data that were not normally distributed were made using the Mann–Whitney test or the Kruskal–Wallis tests with Dunn's post hoc test. Differences were considered significant when *p* < 0.05 and Origin 5 software was used (Microcal Software, Inc.). Skeletal muscle fibers data were obtained from ≥6 cells from at least four different flexor digitorum brevis muscle from four mice. For animal studies, no randomization was used and no blinding was done. The investigators were not blinded to the group allocation during the experiment and/or when assessing the outcome. The variance is similar between the groups that are statistically compared.

## Results

### TRPV1 expressed in HEK-293 cells is activated by volatile anesthetics

**Fig. 1:**
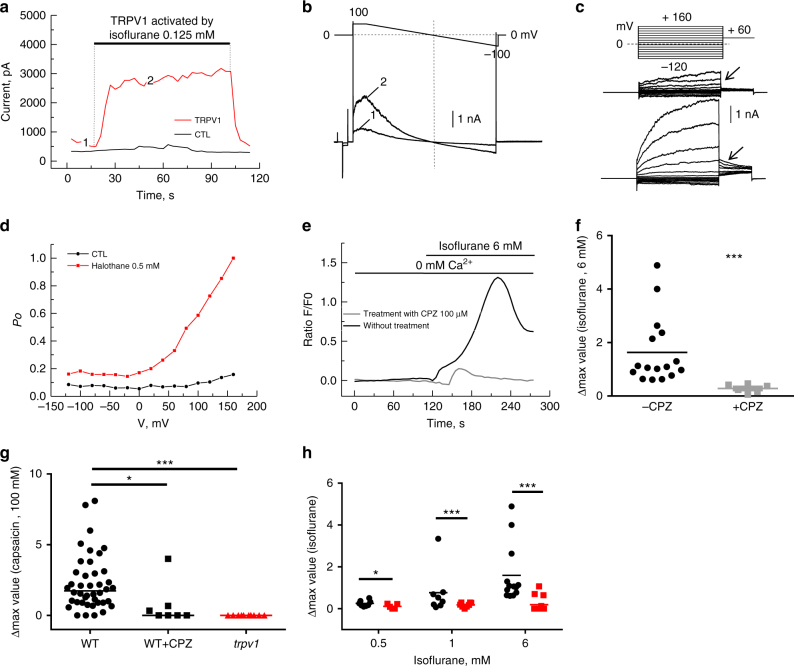
Volatile anesthetics activate TRPV1 channels heterologously expressed in HEK-293 cells and in flexor digitorum brevis (FDB) isolated fibers. (**a**,**b**) Original recordings of the baseline TRPV1 current (I_TRPV1_) (acquired at point 1 of the time course in (**a**)) and isoflurane-activated (0.125 mM) (acquired at point 2 of the time course) in response to pulse protocol with voltage-ramp portion shown above the recordings. Isoflurane activated a membrane current with biophysical properties, such as a prominent outward rectification and close to 0 mV reversal potential, which is similar to the current activated by the established TRPV1 stimulus, capsaicin (data not shown) (*n* = 7). (**c**) Examples of the baseline and halothane-activated I_TRPV1_ in response to the depicted voltage-clamp protocol, which were used to measure voltage dependence of TRPV1 channel open probability (*P*_o_ in (**d**); experiments were performed at 20 °C); arrows in (**c**) point to the I_TRPV1_ tail currents at + 60 mV. Tail currents were measured during the first millisecond of the final step +60 mV and normalized to the maximal tail current. Normalized amplitude as a function of conditioning depolarizing pulse (ranging from –120 to +160 mV) corresponds to the apparent *P*_o_ (mean ± S.E.M.,*n* = 3). Membrane currents were recorded in the whole cell configuration using the Axopatch 200B amplifier (Molecular Devices, Union City, CA). Extracellular solution containing (in mM): 150 NaCl, 1 MgCl_2_, 5 glucose, 10 HEPES, pH 7.3. Intracellular solution containing (in mM): 150 NaCl, 3 MgCl_2_, 5 EGTA, 10 HEPES, pH 7.3. (**e**) Traces show representative curve obtained after stimulation of single fibers with isoflurane (6 mM; black line) or in presence of capsazepine (CPZ; 100 μM; light gray line). (**f**) Changes in fluorescence ratio F/F0 (peak-resting) induced by drugs as indicated in table above graphs. Capsazepine was added 25 min prior to isoflurane. Corresponding scatterplots of Δ max expressed as median; data are from 16 cells (without CPZ) and 6 cells (with CPZ) from at least 4 independent fibers preparations. Changes in fluorescence ratio F/F0 (peak-resting) induced by (**g**) capsaicin (100 μM) or by (**h**) isoflurane (0.5 mM; 1 mM; 6 mM) in C57Bl6J (black) or *trpv1*^−/−^ mice (red). Corresponding scatterplots of max value expressed as median; for the capsaicin response, data are from 42 cells (wild-type, WT), 7 cells (WT + CPZ), and 10 cells (*trpv1*^−/−^) from at least 4 independent fibers preparations. For the isoflurane response, data are from 6 and 5 cells (0.5 mM isoflurane, WT and TRPV1^−/−^ respectively); 9 and 8 cells (1 mM isoflurane, WT and TRPV1^−/−^ respectively); and 12 and 10 cells (6 mM isoflurane, WT and TRPV1^−/−^ respectively) from at least 4 independent fiber preparations. Mann–Whitney tests were used: *p < 0.05, **p < 0.01, ***p < 0.001, ****p < 0.0001

First, to assess the clinical relevance of studying TRPV1 in MH, we tested the effects of volatile anesthetics (VAs) on TRPV1 activity expressed in HEK-293 cells. Whole-cell patch-clamp experiments showed that isoflurane activated TRPV1 (Fig. 1a,b). No currents were observed in similar condition in untransfected HEK-293 cells as well as in cells transfected with pEGFP-N1 alone (CTL condition; see Materials and methods section). Next, we asked how VAs activate TRPV1. To address this, we hypothesized that VAs sensing could be tightly linked to voltage-dependent sensing as it has been demonstrated for temperature or the chemical agonist capsaicin.^22^ We investigated this possibility by performing the same patch-clamp protocol that has been used to demonstrate that the agonist capsaicin functions as a gating modifier shifting activation curves toward physiological membrane potentials.^22^ Comparison of the steady-state activation curves of the background membrane current carried through TRPV1 channels (ITRPV1) in HEK-293 cells with or without exposure to halothane showed that anesthetic produces a depolarizing shift in the voltage dependence of the TRPV1 channel activation by about 100 mV (Fig. 1c,d), which may underlie its agonistic action mechanism.

### TRPV1 is a SR Ca^2+^ channel activated by VAs in skeletal muscle cells

We have previously shown that Trpv1 is a functional SR Ca^2+^-leak channel in adult mouse skeletal muscle cells.^14^ The question remained as to whether endogenous Trpv1 could be involved in halothane-induced release of Ca^2+^ from internal stores in skeletal muscle. We tested this possibility on skeletal muscle cells isolated from C57BL6J mice flexor digitorum brevis (FDB) muscle in an external Ca^2+^-free solution. Fig. 1e shows that Fluo-4-loaded cells undergo massive increase of cytosolic [Ca^2+^] after isoflurane perfusion (median of Δmax = 1.09; *n* = 29) (Fig. 1e,f). Interestingly a pretreatment with capsazepine (CPZ), an inhibitor of Trpv1 significantly reduces isoflurane-induced Ca^2+^ release (median of Δmax = 0.3; *n* = 6), suggesting that muscle response to isoflurane is linked with Trpv1 activation. To confirm these results, we repeated those experiments in *trpv1*^−/−^ mice muscle cells. As expected for these cells, the response to capsaicin (100 µM) was not observed (Fig. 1g). Next a dose-dependent response to isoflurane on SR Ca^2+^release in WT and*trpv1*^−/−^ muscle cells was tested. Isoflurane’s maximal effect was obtained at a concentration of 1 mM isoflurane in WT cells, but in the same conditions a clear reduction in *trpv1*^−/−^ muscle cells' Ca^2+^ release was recorded. This strongly suggests that SR Ca^2+^-store release in differentiated skeletal muscle cells stimulated by isoflurane is mediated by the Trpv1 channel (Fig. 1h).

### Identification of *TRPV1* rare variants from patients who suffer from malignant hyperthermia

**Fig. 2:**
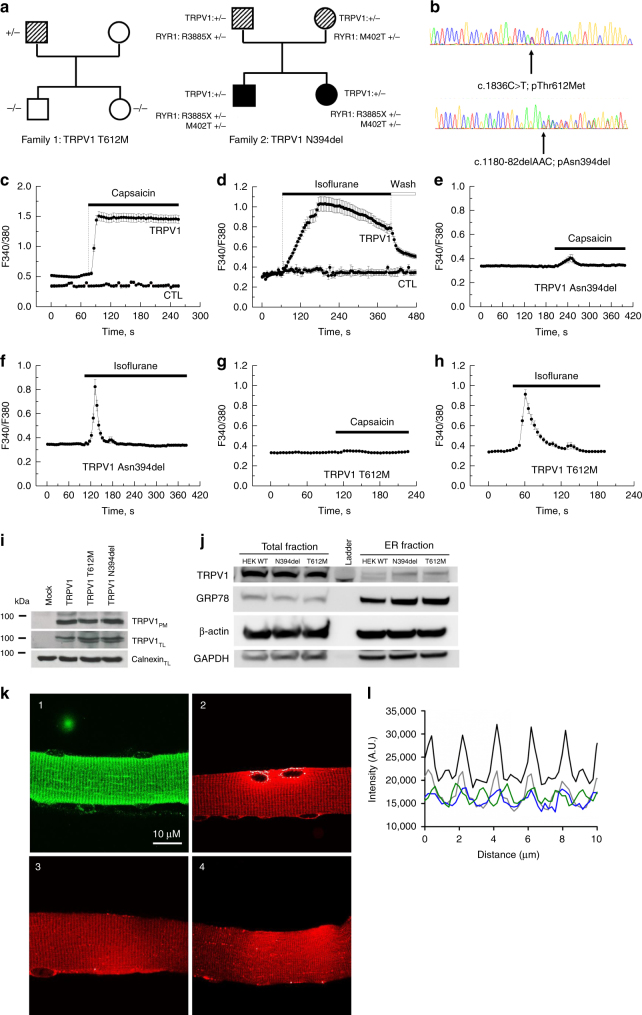
*TRPV1* variations from patients who suffer from malignant hyperthermia. (**a**) Segregation of*TRPV1* variations. Individuals tested MHEh are depicted with hatched symbols. Black symbols refer to patients diagnosed with a congenital myopathy and MH sensitivity. Arrows indicate probands referred to the laboratory for genetic studies according to initial diagnosis: malignant hyperthermia crisis for family 1 and myopathy for family 2. Family 1: the proband was referred to genetic investigation for postoperative hyperthermia after anesthesia. No significant variant was found in the *RYR1* gene sequence. The c.1836C>T; pThr612Met variation was found in targeted analysis of the gene. Family 2: the proband was referred to genetic investigation for muscle weakness. He responded positively to the in vitro contracture test (MHS) and further familial studies were undertaken for MH sensitivity. Two mutations in the *RYR1* gene (c.1205T>C;p.Met402Thr and c.11653C>T;p.Arg3885X) were found inherited from each of the parents in the proband and his symptomatic sister’s DNA. The haplo insufficiency that was revealed during sequencing of the father *RYR1* messenger RNA (mRNA) cannot account for its MH sensitivity to halothane in the in vitro contracture test (MHSh). The c.1180-82delAAC; pAsn394del variant was found in a targeted analysis of the*TRPV1* gene. The variation was also found in his daughter’s DNA. (**b**) Electropherograms from DNA sequencing showing*TRPV1* variation c.1835C>T; pThr612Met and c.1180-82delAAC; pAsn394del respectively for family 1 and 2. (**c**–**h**) Ca^2+^ imaging experiments were performed using the cytosolic Ca^2+^ probe fura-2 (at 37 °C). Representative time course of cytosolic Ca^2+^ concentration following capsaicin (10 µM) or isoflurane (0.5 mM) exposure in HEK-293 cells transfected with the wild-type TRPV1 or the TRPV1 mutants. Each experiment was repeated three times (field of 35–45 cells) and representative experiments are presented (mean ± SE). (**i**) Cell-surface biotinylation analysis of TRPV1 wild-type and mutants transfected cells. The empty vector was used as a control (mock). TRPV1 expression was analyzed by immunoblotting for the biotinylated plasma membrane fraction (TRPV1PM) or total cell lysates (TRPV1TL) and calnexin was used as a loading control. (**j**) Endoplasmic reticulum (ER) enrichment of WT HEK or transiently transfected with TRPV1 N394del or T612M and total fraction. GRP78 is used as an ER loading control, glyceraldehyde 3-phosphate dehydrogenase (GAPDH) for the cytosol compartment, and B-actin as a general loading control. (**k**,**l**) Patterns of expression of human or mutant (T612M and N394del) TRPV1 transfected in flexor digitorum brevis (FDB) muscle fibers of *trpv1*^−/−^ mice. Representative confocal images of immunofluorescence labeling of (1) Trpv1, (2) human TRPV1-mcherry, (3) TRPV1 T612M-mcherry, and (4) TRPV1 N394*del-*mcherry. (**l**) Average intensity profiles superposed: native Trpv1 (black line), hTRPV1-mcherry (gray line), TRPV1 T612M- mcherry (blue line), and TRPV1 N394del-mcherry (green line)

To date, *TRPV1* has not been clearly linked to any hereditary disease, but we hypothesized that TRPV1 channel may be involved at a genetic level in MH, because it is well established that other MH-susceptible genes than *RYR1* exist, and that TRPV1 can be activated by volatile anesthetics and mediate Ca^2+^ release in skeletal muscle. We sequenced the*TRPV1* gene in a cohort of 28 MH patients who had been previously shown to have enhanced in vitro sensitivity to halothane in a presymptomatic test relying on the contraction developed by a skeletal muscle biopsy upon either caffeine or halothane stimulation (in vitro contracture test, IVCT), which is the gold standard for screening MH-susceptible patients. Because muscle biopsies from patients with *RYR1* mutations trigger strong contractions in the presence of both caffeine and halothane, we focused our search on patients showing only hypersensitivity to halothane, and not caffeine, during IVCT. These patients are referred to as MHSh. Two rare genetic variations in the *TRPV1* gene were found in two independent patients (Fig. 2a, b). The first variant corresponded to a missense variation (c.1836C>T;p.Thr612Met), leading to the substitution of threonine 612 by a methionine (T612M), and was identified in a patient who developed postoperative hyperthermia after anesthesia. The patient was tested MHSh several months after the event. The second variant (c.1180-82delAAC;p.Asn394del) corresponded to the inframe deletion of three nucleotides leading to the deletion of asparagine 394 (N394del) in an asymptomatic patient tested MHSh during a family study. The *RYR1* gene of the two patients was completely sequenced and only one displayed a significant change in the *RyR1* sequence. The patient with the N394del variant was also carrier of a nonsense *RYR1* mutation in the heterozygous state. This mutation leading to a premature stop codon in one allele of the gene could however not account for a MHS phenotype. Indeed, MH has a dominant mode of inheritance and a pathophysiology related to a hypersensitive RyR1 channel only caused by missense or inframe deletion variants. The T612M variation was found in the databases (rs199539626) with a minor allele frequency (MAF) of 0.0013 (ExAC browser), and classified as tolerated or benign by SIFT and Polyphen prediction software. The N394del was not reported, but the Asn in the 394 position was found to have mutated to Ser in only one allele in the ExAC database. Overall, the two *TRPV1* variants were either unknown or below a low MAF threshold, as compatible with rare and triggered diseases such as MH.

### Impact of the *TRPV1* variants on Ca^2+^ homeostasis

We characterized the impact of the two *TRPV1* variants on Ca^2+^ homeostasis by performing Ca^2+^ imaging experiments in HEK-293 cells. We found that both *TRPV1* variants display a fast and transient Ca^2+^ response to isoflurane exposure compared with wild-type *TRPV1* transfected cells (CTL) (Fig. 2c–h). Importantly, capsaicin does not alter [Ca^2+^]i in cells transfected with the variant. We checked that the same level of TRPV1 proteins (wild-type or variants) were expressed at the plasma membrane as detected by cell-surface biotinylation assays (Fig. 2i). We also confirmed similar level of total fraction and ER enrichment of TRPV1WT, TRPV1 N394del, and TRPV1 T612M transiently transfected in HEK-293 (Fig. 2j). This suggests that the modification of the channel activity is not due to an altered trafficking of the mutated channels.

### *TRPV1* variants transfected in skeletal muscle cells are highly sensitive to VAs

We evaluated the consequences of the *TRPV1* variants on SR Ca^2+^-leak in skeletal muscle. For this, we transfected *trpv1*^−/−^ FDB muscles with plasmids coding for the wild-type or mutants human TRPV1 (T612M or N394del). Previously, we localized native mouse Trpv1 in the longitudinal part of SR in skeletal muscle fibers^14^ as illustrated in Fig. 2k. We confirmed with tagged versions of the human or mutant TRPV1 that they were also expressed in longitudinal SR (Fig. 2, panels 2, 3, 4). Intensity profiles clearly show single peaks repeated every 2 µM, similar to the Trpv1 native profile previously obtained in mouse skeletal cells.^14^ Thus, human Trpv1 and its mutated forms seem to localize in the longitudinal part of SR (Fig. 2k, l). In accordance with our previous results, we did not notice any plasma membrane labeling.

**Fig. 3:**
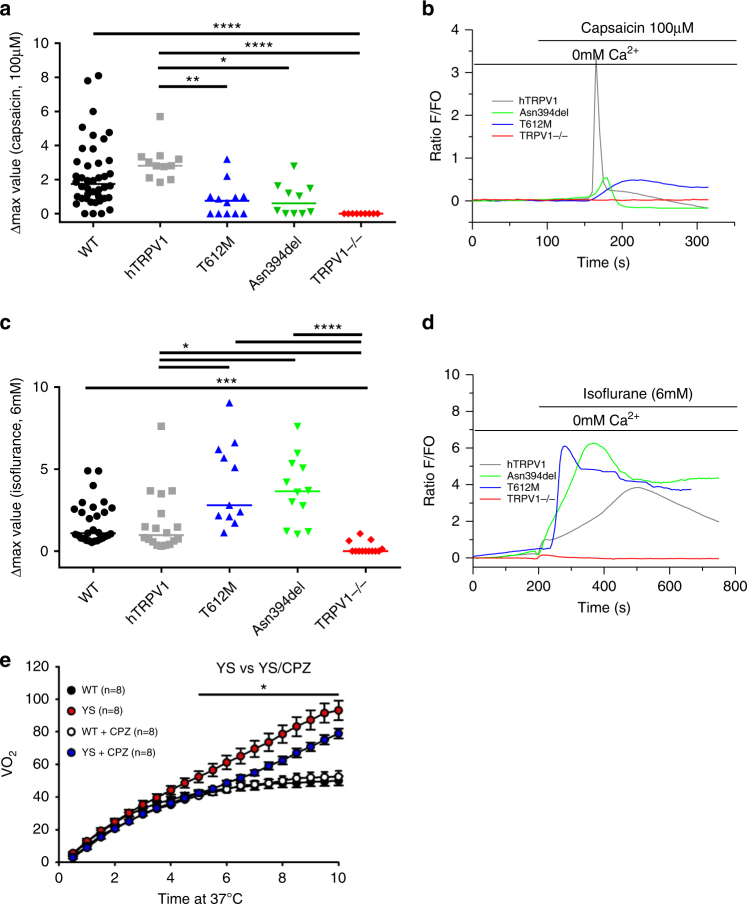
Decrease in capsaicin sensitivity and increase in isoflurane sensitivity in mutants *TRPV1* (T612M; N394del) transfected in FDB muscles fibers of *trpv1*^−/−^ mice and effect of Trpv1 inhibition by capsazepine on heat-induced sudden death in Y524S mice heat challenged at 37 °C. Changes in fluorescence ratio F/F0 (peak-resting) induced by (**a**,**b**) (100 μM) capsaicin or (**c**,**d**) isoflurane (6 mM) in C57Bl6J (black), TRPV1^−/−^ (red), humanTRPV1-mcherry (gray), TRPV1 T612M-mcherry (blue), or TRPV1 N394*del*-mcherry (green). Corresponding scatterplots of max value expressed as median. For the capsaicin response, data are from 42 cells (WT), Eleven cells (hTRPV1), 12 cells (T612M), 10 cells (N394del), and 9 cells (TRPV1^−/−^) from at least 4 independent fiber preparations. For the isoflurane response, data are from 29 cells (WT), 18 cells (hTRPV1), 11 cells (T612M), 12 cells (N394del), and 13 cells (TRPV1^−/−^) from at least 4 independent fiber preparations. Kruskal–Wallis tests with Dunn's post hoc tests were performed: *p < 0.05, **p < 0.001, ***p < 0.0001, ****p < 0.0001. (**e**) Oxygen consumption (VO_2_) during a 15 min exposure of mice at 37 °C in Y524S RYR1 mouse model (YS) or in WT mouse (WT). *n* = 8 for each condition

To assess the role of TRPV1 mutants in the SR Ca^2+^ response to general anesthetics or capsaicin, we compared the sensitivity to isoflurane and capsaicin of WT mouse Trpv1 (C57BL6J) and human TRPV1 (hTRPV1), TRPV1, T612M, or TRPV1 N394del expressed in the *FDB* fibers of *trpv1*^−/−^ mice. Nontransfected cells of *trpv1*^−/−^ mice were used as a negative control. Cells were loaded with Fluo-4 AM to measure the increase in cytosolic Ca^2+^ level due to SR Ca^2+^ release. As illustrated by Fig. 3a, b, except for the nontransfected *FDB* muscle fibers of *trpv1*^−/−^ mice, capsaicin (100 µM) induced an increase in cytosolic Ca^2+^ level. Capsaicin response was similar in endogenous mouse Trpv1 and hTRPV1 (median of max value = 1.74; *n* = 42 and 2.81; *n* = 11 respectively). Nevertheless, we noticed a significant decrease in amplitude of capsaicin response in cells expressing T612M variant (median of max value = 0.76; *n* = 12) and in cells expressing N394del variant (median of max value = 0.61; *n* = 10).

We further measured the amplitude of isoflurane response of these constructs (Fig. 3c, d) and found no differences between endogenous mouse Trpv1 and human TRPV1 (median of max value = 1.09; *n* = 29 and median of max value = 0.98; *n* = 18 respectively). Interestingly, the amplitude of isoflurane response in *trpv1*^−/−^ cells expressing T612M and N394del variants was significantly higher (2.80; *n* = 11 and 3.65; *n* = 12 respectively). The delay and time to peak of isoflurane responses were similar (data not shown). Altogether, these experiments strongly suggest that the T612M and N394del variants of TRPV1 have an altered channel function and are in particular much more sensitive to isoflurane that the wild-type channel.

On the basis of our previous experiments, we hypothesized that the TRPV1 channel could be an important part in the pathophysiology of MH triggering. We reasoned that TRPV1 activation could either directly result in massive Ca^2+^release whenever mutations enhance its sensitivity to VAs, or that initial Ca^2+^ release by TRPV1 stimulated by VAs could trigger or enhance RYR1-mediated Ca^2+^ release. To explore this hypothesis, we investigated the role of Trpv1 in the phenotype of an MH mice model, the mice line with a knocked-in Y524S mutation in Ryr1 channel. These mice bear a mutation analogous to the Y522S mutation that is associated with MH in humans,^23^ and die after exposure to 37 °C for longer than 15 min. The Y524S mice display a dramatic heat-induced hypermetabolic response when exposed to 37 °C compared with WT mice that can be measured by VO_2_ consumption. We showed that treatment with the Trpv1 antagonist capsazepine significantly slows down the heat-induced hypermetabolic response in this mouse model (Fig. 3e). This suggested that Trpv1 may be contributing to the mechanism underlying the hyperthermia response of this Y524S Ryr1 model. These findings suggest that TRPV1 and related mutants could be a new therapeutic target for treating muscle diseases due to altered regulation of Ca^2+^ release.

## Discussion

In the present study, we show that Trpv1 functions as a Ca^2+^-release/Ca^2+^-leak channel in adult skeletal muscle in response to VAs exposure. TRPV1 is a well-known polymodal cellular sensor for heat and other physiological stimuli.^24–27^ However, how this channel is activated by diverse physical and chemical stimuli remains largely unknown. Recently, we demonstrated that anesthetic produces a depolarizing shift in the voltage dependence of the TRPM8 channel activation,^28^ which might underlie its agonistic action mechanism. Our results suggest that TRPV1 shares a similar behavior, because VA exposure also produces a depolarizing shift in the voltage dependence of TRPV1.

Here, we also identified a new role of TRPV1 in a human pathology and we proposed it as a new therapeutic target. We have also identified, for the first time, two *TPV1* mutations in patients suffering from a human pathology and, to our knowledge, TRPV1 has never been involved before in any genetic disorders. Indeed, regarding our previous data^14^ and the fact that we identified VAs as potent activators of this channel in skeletal muscle cells, we hypothesized that TRPV1 could be part of malignant hyperthermia (MH) crisis. MH is a triggered muscle disease and is known to be a hereditary disease. Moreover, Ca^2+^ signaling is so far known to be linked to different muscle pathologies such as MH. For this reason, the regulation of intracellular Ca^2+^ signaling is an area of intense research to better understand muscle pathophysiology. Concerning MH, the pathogenic implications of different mutants of the main intracellular Ca^2+^ channel, RYR1, are well accepted in MH, where the release of Ca^2+^ through an abnormal RYR1 in the presence of volatile anesthetic activates an uncontrolled increase in Ca^2+^ release. This increase in cytosolic Ca^2+^ leads to the hypermetabolic response characteristic of MH. Although central for MH susceptibility, RYR1 mutations do not constitute the exclusive genetic cause for this pathology. Accordingly, two*TRPV1* variants were discovered in patients who tested MH sensitive. The first *TRPV1* variant (T612M) was found in a patient with a postoperative hyperthermia after anesthesia. The second variant (N394del) was discovered in an asymptomatic patient. The two variations were either unknown or with very low prevalence in human genetic databases, as compatible with a triggered pathology such as MH, but genetic evidence that these *TRPV1* variations were responsible for MH was difficult to obtain for these cases. In the second family (N394del),*RYR1* compound heterozygous mutations led to myopathy in the two children. Moreover, family members studied were limited, thus it is difficult to clearly establish a genotype to phenotype correlation.

We have also shown previously that Ca^2+^ release in skeletal muscle stimulated by the TRPV1 agonist capsaicin was a two-phase process consisting of a first step of Ca^2+^ release that is dantrolene resistant and a second step that is inhibited by dantrolene, which is a known inhibitor of RYR1 function.^14^ This suggested that Ca^2+^ released directly by TRPV1 from the SR could in turn activate a RYR1-mediated Ca^2+^ release. Therefore, TRPV1 could also directly be related to RYR1 function as an intracellular channel able to prime massive Ca^2+^ release by RYR1. To check this hypothesis, we performed in vivo experiments in transgenic mice expressing a ryr1 mutation responsible for MH.^23^ As already described, environmental heat induces increase in body temperature of these animals and a hypermetabolic response that can lead to death. A pretreatment of the mice with capsazepine, an inhibitor of Trpv1, significantly decreased the hypermetabolic crisis, suggesting that Trpv1-mediated Ca^2+^ release at least participates to the pathophysiology of this MH mouse model. Altogether, our data highlight the potential role of TRPV1 in skeletal muscle Ca^2+^ homeostasis. It raises the possibility that mutations in TRPV1 could induce uncontrolled Ca^2+^ release, either directly raising intracellular Ca^2+^ concentration above a threshold sufficient to trigger MH or able to trigger RYR1-mediated Ca^2+^release that will reach this threshold. Accordingly, we propose a new mechanism in MH pathogenesis induced by an initial reticular Ca^2+^ release through endogenous TRPV1 channels activated by volatile anesthetics. Our data also suggest that Ca^2+^ mobilization occurring through a channel composed of TRPV1 would represent a dantrolene-resistant step in RYR1 activation by drugs during MH. Dantrolene represents the only drug to reverse anesthetic-induced MH episodes approved by the US Food and Drug Administration (FDA); however, because it is not always effective, other drugs are needed. Finally, TRPV1 could represent a new therapeutic target for treating various myopathies characterized by a key altered regulation of Ca^2+^release. This study also uncovers the possibility that TRPV1 mutants may contribute to various muscle phenotypes related to Ca^2+^ homeostasis.

In conclusion, our study provides crucial understandings concerning the SR Ca^2+^-release/Ca^2+^-leak mechanism and highlights a potential actionable therapeutic target, thereby opening up a new avenue of research into the physiology and physiopathology of skeletal muscle.
